# What Rural Women Want the Public Health Community to Know About Access to Healthful Food: A Qualitative Study, 2011

**DOI:** 10.5888/pcd13.150583

**Published:** 2016-04-28

**Authors:** Leslie R. Carnahan, Kristine Zimmermann, Nadine R. Peacock

**Affiliations:** Author Affiliations: Kristine Zimmermann, Center for Research on Women and Gender, and School of Public Health, University of Illinois at Chicago, Chicago, Illinois; Nadine R. Peacock, School of Public Health, University of Illinois at Chicago, Chicago, Illinois. Ms Carnahan is also affiliated with the School of Public Health, University of Illinois at Chicago, Chicago, Illinois.

## Abstract

**Introduction:**

Living in a rural food desert has been linked to poor dietary habits. Understanding community perspectives about available resources and feasible solutions may inform strategies to improve food access in rural food deserts. The objective of our study was to identify resources and solutions to the food access problems of women in rural, southernmost Illinois.

**Methods:**

Fourteen focus groups with women (n = 110 participants) in 4 age groups were conducted in a 7-county region as part of a community assessment focused on women’s health. We used content analysis with inductive and deductive approaches to explore food access barriers and facilitators.

**Results:**

Similar to participants in previous studies, participants in our study reported insufficient local food sources, which they believe contributed to poor dietary habits, high food prices, and the need to travel for healthful food. Participants identified existing local activities and resources that help to increase access, such as home and community gardens, food pantries, and public transportation, as well as local solutions, such as improving nutrition education and public transportation options.

**Conclusion:**

Multilevel and collaborative strategies and policies are needed to address food access barriers in rural communities. At the individual level, education may help residents navigate geographic and economic barriers. Community solutions include collaborative strategies to increase availability of healthful foods through traditional and nontraditional food sources. Policy change is needed to promote local agriculture and distribution of privately grown food. Understanding needs and strengths in rural communities will ensure responsive and effective strategies to improve the rural food environment.

## Introduction

Access to affordable, culturally suitable, and high-quality food within a reasonable distance of a residence has a positive association with more healthful diets in the United States (1,2). Meeting the recommended daily intake of fruits and vegetables is associated with reduced risk among adults for several chronic diseases such as diabetes, hypertension, and cardiovascular diseases (2–5). However, residents of the rural United States may not meet the recommended fruit and vegetable intake for numerous reasons related to food access (6). Barriers to food access in rural areas are well-documented and include high costs and long-distance travel to retailers that sell fresh produce (1,2,7). Compared with urban food deserts, rural food deserts may have fewer retail options, less food variety, and fewer resources to address food security (8,9).

Most scientific literature focuses on barriers for rural populations, not on perceived solutions or resources (1,2,7). Several programs emerged to improve food access for urban communities, but the formative work for these programs drew largely on the perceptions of urban residents and may not translate to rural settings (10–13). Our study was designed to address these gaps in the literature. The objective of our study was to identify resources and solutions to the food access problems of women in rural, southernmost Illinois.

## Methods

We used an assets-focused (resources and solutions) approach to analyze data generated by focus groups in which women identified resources and solutions. We defined *resources* as existing factors that promote food access and *solutions* as proposed or desired factors that promote food access. Women’s perceptions about food access are important because women are often responsible for household food purchases and preparation decisions (14,15). Understanding perspectives and contextual factors is an important step in developing public health solutions to address rural communities’ needs.

**Setting.** The Southern Seven Health Department, which serves the rural southernmost 7 (S7) counties in Illinois, identified obesity, diabetes, cardiovascular disease, and cancer as priority health areas (16). With higher rates than Illinois overall, 70.6% of S7 residents are overweight or obese (63.7% in Illinois), 11.7% were told they have diabetes (10.2% in Illinois), 34.4% were told they have high blood pressure (30.1% in Illinois), and 46.6% were told they have high cholesterol (36.6% in Illinois) (17). In S7 counties, only 15.4% of adults reported meeting the recommended daily fruit and vegetable intake (22.6% in Illinois) (17). The high rates of chronic disease may be attributed in part not only to unhealthful dietary habits but also to lack of food access. Each S7 county has at least one census tract identified as a food desert (9).

**Focus group sample and recruitment.** Fourteen focus groups were conducted as part of a comprehensive community health assessment to identify and examine women’s health issues in the S7 counties and to inform development of the region’s health programs for women (18). We recruited women with diverse sociodemographic characteristics (age, race, residence, and socioeconomic status) by using flyers, announcements in church, community newsletters, and newspaper advertisements. Those who met age and residency requirements were invited to attend focus groups in their communities. Before the start of each focus group, informed consent was obtained from each participant. The University of Illinois at Chicago institutional review board approved this research.

**Focus group procedures.** Focus groups were conducted in all S7 counties in February and March 2011; they took place in public locations such as clinics, churches, hospitals, a library, and senior-living facilities. Health educators from the local health department were trained as facilitators by the academic partner. The facilitators used a semistructured guide (Appendix) to elicit perceptions of the communities’ and women’s health needs. Scripted questions asked for respondent’s ideas about the biggest health problems and needs in the community, ways to address the community’s health issues, and strengths in the community that help people stay or become healthy. Questions were open-ended, but interviewers sometimes suggested categories of health issues, solutions, or strengths to stimulate respondents’ thinking if participants did not offer suggestions on their own. The focus group facilitators were trained in facilitation methods and therefore, at their discretion, used additional probes not included in the focus group guide to encourage discussion. They encouraged natural conversation but also used probes to stimulate detailed responses and to manage discussions so that all topics were covered within one hour. Each participant received a $15 gift card as an incentive. Focus group discussions were audiorecorded and transcribed shortly after completion. All transcripts were sent to the facilitators to verify accuracy and provide clarification. Before analysis, we verified each transcript against its audio file.

**Research team and analysis procedures.** We conducted a secondary analysis of the focus group data to glean additional public health information. We used inductive content analysis to examine the patterns and themes in our data and develop a theory related to perceptions about health among participants. We used deductive content analysis to explore food access barriers and facilitators and used published data on food access to inform our conceptual framework; our data were used for theory confirmation. Although our analysis was grounded in the data, we had a predetermined conceptual framework that reflected the original focus group interview guide, which included broad questions related to how rural S7 women think and talk about health and related behaviors. Two researchers, both trained in qualitative research methods, conducted the analysis and interpretation of focus group data. A third senior qualitative researcher provided oversight and guidance. We first reviewed and annotated all 14 transcripts, recording initial thoughts about content relevant to the research questions. This review informed the development of a set of codes used in a systematic analysis facilitated by qualitative data analysis software (ATLAS.ti, version 7; Scientific Software Development GmbH). We documented our analysis by using a detailed audit trail. Then, using principles of constant comparison analysis, we expanded our codebook to encompass emerging themes in the narrative data (19). A subset of transcripts was first coded by both team members. We then discussed coding discrepancies and revised our codebook to clarify concepts and code definitions. The process was repeated multiple times to create the final codebook. Our final codebook consisted of 45 codes, separated into 8 coding families: access, demographics, environment, food, health, heath care, illness and disease, and physical activity.

Using the finalized codebook, we independently coded segmented transcripts to calculate crude agreement and Cohen’s κ. The process was repeated multiple times until we reached an acceptable agreement score (crude agreement = 98.1%; mean κ = 0.89). After reaching agreement, one researcher coded the remaining transcripts. During team meetings, we identified broad topic areas for additional exploration. We then selected codes relevant to those topic areas, and used ATLAS.ti’s search function (Query Tool) to retrieve relevant text passages (Quotations). We independently reviewed the queries and recorded detailed interpretive memos. During regular team meetings, we discussed our preliminary findings and ran additional queries to confirm and establish themes.

## Results

A total of 110 women participated in the 14 focus groups (Table 1); the mean number per group was 7 participants (range, 3–13 women). Seventy-nine (71.8%) participants were white and 31 (28.2%) were African American. Thirty percent of participants (n = 33) were aged 70 or older.

**Table 1:** Demographic Characteristics of Focus Group Participants (n = 110), Study Among Women on Access to Healthful Foods in the 7 Southernmost Counties of Illinois, 2011

Characteristic	n (%)
**Race**	
African American	31 (28.2)
White	79 (71.8)
**Ethnicity**	
Hispanic	2 (1.8)
**Age group, y**	
18–30	26 (23.6)
31–50	24 (21.8)
51–70	27 (24.6)
≥70	33 (30.0)
**County of residence**	
Alexander	27 (24.5)
Johnson	9 (8.2)
Massac	9 (8.2)
Pope or Hardin^a^	31 (28.2)
Pulaski	18 (16.4)
Union	16 (14.5)

a Because of small population sizes, the focus groups in Pope and Hardin counties were combined.

Participants identified physical, economic, and social barriers to food access, particularly in acquiring foods they described as healthful (Table 2). Participants indicated that their communities had an insufficient number of grocery stores and that travel times to stores that sold affordable and high-quality foods, such as fresh fruits and vegetables, were excessive. Some women discussed alternatives to grocery stores, such as gas stations, convenience stores, and fast-food restaurants; however, they indicated the alternatives often lacked healthful choices. Low-income status was also identified as a barrier to healthful food access. Being poor exacerbated challenges to local food acquisition and travel outside one’s community to access healthful foods. Broader societal norms also affected access. Because of multiple responsibilities in the household and working outside of the home, women had limited time and energy. Because of competing priorities, some were unable to spend time on meal preparation and relied on fast food and eating outside the home.

**Table 2:** Sample Quotations on Barriers to Healthful Food Access, by Subtheme, From Focus Group Participants (n = 110), Study on Access to Healthful Foods in the 7 Southernmost Counties of Illinois, 2011

Subtheme, County, Age Group	Comment
**Physical barriers**	
Pope or Hardin,^a^ ages 31–50 y	We have one grocery store here that you can get produce from, you know, we have no access. You have to drive . . . 45 minutes away to get good produce. So, things that are . . . healthy, we don’t have access to, immediate access to.
**Economic barriers**	
Pope or Hardin,^a^ ages 18–30 y	We can only afford to go to the grocery store, you know, once every 2 weeks or so. So it’s not like you’re getting fresh produce, it’s not like you can keep it in the house that long.
Union, ages 31–50 y	Fresh fruits and vegetables and things like that are so much more expensive and if families can’t afford it, then they’re buying the processed foods that are so much cheaper.
**Social barriers**	
Massac, ages 51–70 y	You know people’s lifestyle has changed. Women work out of the home, when they used to stay home and prepare better meals. Now we all go out to eat because more women work outside of the home.
Alexander, ages ≥70 y	A lot of people don’t have relatives that will take them to the grocery store, or friends, then they’ve got to rely on other people, and pay, a lot.
**Other barriers**	
Alexander, ages 31–50 y	Participant 1: I mean, I have gone to the grocery stores, and I looked at vegetables, and I’m like, “They’re selling this?” Participant 2: Well, it’s moldy! You can actually find mold. Participant 1: It’s awful.
Pulaski, ages 18–30 y	You have like fruits and stuff like apples, they bruise, bananas, they get ripe after like 2 weeks, or you can buy like a thing of Oreos, they’ll last you a few months . . . and then the Oreos cost less than the apples and stuff so they last longer.

a Because of small population sizes, the focus groups in Pope and Hardin counties were combined.

Participants also described numerous existing resources and proposed solutions to food access challenges (Table 3). Personal, community, and school gardens were described as both existing resources and proposed solutions to increase food access, because they were seen as a way to fill gaps left by local grocery stores by providing fresh and attractive foods for consumers. Some participants with home gardens described sharing garden bounty with friends, neighbors, and other community members, and others stated that they regularly preserved food from their gardens so that they had access to these foods when gardening was not feasible. They suggested community classes about gardening, food preservation (eg, canning), and quick and healthful food preparation as solutions to increase local access to healthful food. One participant suggested setting up a market for community members to distribute and sell excess produce.

**Table 3:** Existing Resources and Proposed Solutions at the Individual, Community, and Policy Levels to Eliminate Barriers to Healthful Food Access: Sample Quotations from Study on Access to Healthful Foods in the 7 Southernmost Counties of Illinois, 2011

Level	Subtheme^a^	Quotes Representative of Subtheme^b^
Individual	Education about shopping on a budget, nutrition, and food preparation and preservation (R, S) Personal gardens (R, S)	A teaching or a cooking class [to teach people how to cook in a healthful way]. [Pope or Hardin,^c^ ages 18–30] Participant 1: And fruit is so expensive . . . you can’t afford to go to the store. Participant 2: You could grow your own. [Pulaski, 51–70]
Community	Farmers markets for community members to purchase food, and to sell bounty from garden (R, S) Faith- and community-based organizations that provide meals (R) Transportation services and meal delivery services (R) Food banks (R) Form coalitions to address regional food access issues (R) Community- and school-based gardens (R, S) Meal sharing with neighbors (S)	[The health department] used to put out a resource booklet . . . with everything that you want to know, [food] pantries . . . and all that stuff. [Alexander, ages 31–50 y] And our food pantry actually delivers to, you know, 30 to 40 different shut-ins. Because these elderly folks can’t get out, even to get the assistance that they need, grocery-wise. You know, they just can’t. They either don’t have family or don’t have, you know, whatever. [Pulaski, ages 51–70 y] Churches, give a lot of um, helping the poor, as far as food banks and giving them money. . . . This community [is] . . . very close knit when it comes to pulling together for somebody that’s in need. [Pope or Hardin,^c^ ages 31–50 y] Another thing that would be nice is, you know how people have so much food from their garden that they can't use it all that they’re always bringing it somewhere to give it away? Well, if it was an organized, you know, [as a] free market. . . . Even if it was the farmer’s produce that could just go to the farmers market. [Johnson, 51–70 y] I’m by myself, and it doesn’t pay to fix a [meal] . . . for one person. Now, if we were really good neighbors, we’d all pick a day a week and cook for everybody and share. [Alexander, ages ≥70 y]
Policy	Federal food assistance programs (R, S)	You know, I have read this week that people in Congress are voting to not fund WIC anymore. . . . And it’s such a tiny little program compared with some. . . . How many of us call our legislators when we don’t like those things. . . . Besides me? I call. [Massac, ages 51–70 y]

Abbreviations: R, resource (existing); S, solution (proposed).

a Resources and solutions proposed by participants may overlap because of the geographic scope of the focus groups and because some counties may not have a resource that exists in another county.

b Respondent identified by county of residence and age group.

c Because of small population sizes, the focus groups in Pope and Hardin counties were combined.

Participants mentioned 2 mass transit companies that alleviate transportation issues in the S7 region. For homebound people, women described food delivery programs, such as Meals-on-Wheels, that provided services in the region. Several participants suggested that the mass transit services could be expanded to assist residents in accessing grocery stores and supermarkets.

Participants spoke of numerous food access resources and solutions particularly pertinent to low-income populations. Many described a sense of a close-knit community and altruistic actions. For example, faith- and community-based organizations distributed food to people through local foods banks and meal services. Participants suggested that community resources for low-income people should be better publicized to increase community awareness.

Participants also proposed solutions related to civic action and policy change to increase food access in their communities. For example, they suggested that community members form coalitions to reduce problems associated with food access. Participants also suggested voicing concerns with government representatives about the impact of cuts in food supplement and assistance programs.

## Discussion

This study contributes to the limited research on women’s perceptions about access to healthful food and solutions to the problem of limited access to such food in rural communities. Our findings suggest that rural communities such as those in the S7 region in Illinois have insufficient local sources of healthful foods, which contributes to poor dietary habits and the need to travel for healthful foods. This need to travel places a burden on segments of the population who have difficulty accessing transportation (eg, low-income residents). Additionally, working women who are often responsible for shopping and cooking may also be burdened by the lack of local access to healthful foods in their communities, potentially affecting their own dietary habits and those of their family members.

Focus group participants mentioned local activities (eg, gardening and hunting) and local resources (eg, food pantries and public transportation) as ways to help increase food access. However, these resources were perceived as insufficient for meeting all residents’ food access needs because the activities are not appropriate or feasible for all S7 residents and the resources are not accessible in all parts of the S7 region. Therefore, multilevel, coordinated, collaborative strategies are needed to increase access. At the individual level, behavior change interventions and education strategies, both of which have been associated with positive changes in dietary habits, could be implemented to mitigate the geographic and economic barriers to food access (20,21). Education, mentioned as both an existing resource and suggested solution, focusing on topics such as gardening, food preservation, produce shelf life, and healthful alternatives to fresh produce (eg, frozen, canned) could reduce reliance on local stores and reduce the need for frequent travel. Education on quick, easy, and healthful meal preparation using the range of available options and choices may reduce consumption of fast food and improve nutrition habits (22). Federal agencies, such as the US Department of Agriculture and the Centers for Disease Control and Prevention, provide toolkits and resources that can be used for educational purposes. The American Dietetic Association recommends including food and nutrition education in programs that address food insecurity (23). Additionally, because focus group participants acknowledged that S7 residents consume fast foods, increasing knowledge about healthful fast-food options may assist rural residents in making more healthful choices. Collaborative implementation of these strategies in community-based settings can maximize reach.

Improving the food environment, particularly if done in combination with educational strategies, has the potential for even greater impact on rural communities than solitary, individual-level health education strategies. Current community resources in S7 counties include farmers markets, food banks, faith-based and other organizations that provide meals, and community and school gardens. Innovative, collaborative solutions that include grocery stores, gas stations, and convenience stores may help to increase availability of healthful foods at the local level. For example, small stores could work together with a common food distributor to purchase food in bulk at a lower price than they can buy food individually and then sell to customers at a lower price. Additionally, local stores could collaborate with farmers in the region that grow and produce food to offer healthful, locally sourced options. Stores can also commit to offering more healthful items on sale. Working with local stores to encourage the sale of healthful food may be combined with food preparation demonstrations and distribution of healthful recipes. Furthermore, research indicates 1) that rural corner store owners are receptive to stocking healthful food such as fruits and vegetables and 2) that customers are willing to purchase them (24).

Additional strategies to make locally grown and organic foods accessible include collecting and distributing fresh produce, such as garden surplus, through local food pantries. This strategy would require providing equipment to food pantries for handling and storing perishable foods. Previous efforts targeting food pantries increased access and consumption of fruits and vegetables (12). Mobile food pantries may also be considered (10,13). Additionally, innovative food-waste reduction and recovery programs could further support access to food. In these new models, excess food from grocery stores and restaurants are redistributed to food-insecure people (25). Local food growers could also be encouraged to sell fresh and healthful foods at local farmers markets (26). Faith- and community-based organizations, described as resources by focus group participants, can be supported to form programs or coalitions that address food access, because these are often natural resource points and health partners in rural communities (26,27). Policy change related to food access should also be considered (28–30). Policies, codes, and zoning laws should be created, modified, or reviewed to promote the distribution and sale of homegrown food. Doing so may ensure that agriculture operations, food production, and farmers markets are able to operate on a neighborhood scale.

This study has several limitations. First, participants were not selected randomly. Although a broad range of ages was included, participants may not fully represent the diversity in southernmost Illinois. Additionally, some participants worked in health care or social service fields, which may have influenced their perspectives about health needs in the community. Some participants previously participated in an intervention in the community to reduce cardiovascular disease risk, which may have biased their responses about dietary behavior. Additionally, because the focus groups were limited to women, responses may not be generalizable to men or children in the region. Finally, the focus groups were conducted to understand women’s broad perceptions about their health needs and not their perceptions about food access specifically. However, the participants’ discussions about food access suggest that it is an important topic.

Our findings suggest that additional research is needed to assess the landscape of rural food deserts and to learn about food availability, costs, and purchasing behaviors. The impact of community gardens, school-based gardens, and farmers markets in rural communities on food access should also be examined. The utility of alternative food access points, such as gas stations and convenience stores, should be investigated, because these may be feasible alternatives in rural and low-density populations with limited food access. Additionally, the integration of alternatives to fresh options, such as frozen fruits and vegetables, into rural diets could be examined.

Given the rising rates of chronic diseases in the United States, understanding the association between food access and chronic disease may help public health practitioners better understand how to address needs at the community and individual level. We must also consider that the effects of food deserts go beyond the distance required to travel and the availability of foods. The impact of food deserts on food access is also related to social and economic factors. Interventions and policies aimed at increasing food access in rural food deserts may consider these social and economic factors. In addition, understanding communities’ visions of healthful food environments will ensure that future researchers will look for ways to provide affordable, culturally suitable, and high-quality food within a reasonable distance from people’s homes. No one approach can solve the problem entirely. Public health researchers and practitioners must consider a combination of structural, economic, and individual behavior changes to adequately reduce the adverse health effects caused by rural food deserts.

## Appendix. Focus Group Guide

**Ice Breaker Instructions for the Facilitator**

*Before participants arrive, spread the ice breaker pictures on a table. As participants come in welcome them, direct them to the refreshments, and give them instructions for the icebreaker exercise. This will give participants something to do while the women gather. Say to participants, “We are going to do an exercise using the pictures on the table. I would like you to pick up any 2 pictures from the table that represent health to you. Once you find 2 pictures you can take a seat, and we will get started once everyone is seated.”*

**Ice Breaker Exercise (no more than 15 mins)**

To start, I would like to ask each of you to introduce yourself and tell us why you were interested in coming to this meeting. Then show us the pictures you chose and tell us what they mean to you – how they represent health to you. So that we have time to complete the whole discussion I will ask each of you to limit yourselves to 2-3 minutes.

**Healthy Communities and Women’s Health **

Now I would like to get your perspectives about the health and health needs of the Southern Seven population in general and women in particular.

What does health mean to you as a woman? Think about your various roles as a woman – a mother, daughter, wife, grandmother, sister, aunt, friend, etc.

I am going to ask you some questions about your “community.” A “community” can be a group of people that live in the same geographical location, or it can be a group of people with similar interests or characteristics, such as race, age, or occupation. A community can be the people living in a town, members of a church or club, students that attend a particular school – or their parents, people who work at a particular location, people who regularly shop at a particular store, eat at a particular restaurant, or receive services at a particular hospital or clinic. You probably belong to more than one community. For the next part of the discussion, think about one community that you are a part of that is located within the southern seven counties.

[*Clarification for facilitator: we don’t want them talking about online communities.*]

What do you consider to be your community?

What do you think are important characteristics or features of a healthy community?

**Health Needs of the Community**

What do you think are the most significant health needs or health problems in your community?

[*Have the co-facilitator list these on a flip chart.*]

What do you think are the causes of the health problems that you mentioned?

How are women affected by these needs or problems?

Is there anything being done to solve the health problems that you talked about? Could you explain?

Do you think more can be done? What kinds of things can be done?

The Southern Seven Health Department recently identified heart disease, obesity, diabetes and cancer as health conditions that need to be addressed in your region. Do you think these are important issues to be addressed? *[Probe: why or why not?] *

a. What do you think can be done to prevent these health conditions, and/or to help individuals who are affected by these health conditions?

b. Are there specific activities/services that can be targeted to women?

Are there health needs or health concerns that specifically affect young girls or teen girls in your community? *[Probe for detail.]*

[*Have the co-facilitator list these on a flip chart.*]

What do you think are the causes of the health concerns that you mentioned?

**Access to Health Care **

Does everyone in your community have access to health care services that they need? If not, which groups do not have access and why?

**Community Strengths **

What do you see as the strengths in your community that can help people be healthy or stay healthy? *[Prompts: Are there services, organizations, resources, facilities?] *

**Closing**

Before we end, does anyone have anything they would like to add to the discussion? Or, does anyone have questions for me? Thank you for taking the time to participate!
